# Influence of One Hour versus Two Hours of Daily Static Stretching for Six Weeks Using a Calf-Muscle-Stretching Orthosis on Maximal Strength

**DOI:** 10.3390/ijerph191811621

**Published:** 2022-09-15

**Authors:** Konstantin Warneke, Michael Keiner, Martin Hillebrecht, Stephan Schiemann

**Affiliations:** 1 Institute for Exercise, Sport and Health, Leuphana University, 21335 Lüneburg, Germany; 2 Department of Sport Science, German University of Health & Sport, 85737 Ismaning, Germany; 3 University Sports Center, University of Oldenburg, 26129 Oldenburg, Germany

**Keywords:** plantar flexors, stretch training, rehabilitation, immobilization-related strength deficit, physical therapy

## Abstract

Rebuilding strength capacity is of crucial importance in rehabilitation since significant atrophy due to immobilization after injury and/or surgery can be assumed. To increase maximal strength (MSt), strength training is commonly used. The literature regarding animal studies show that long-lasting static stretching (LStr) interventions can also produce significant improvements in MSt with a dose–response relationship, with stretching times ranging from 30 min to 24 h per day; however, there is limited evidence in human studies. Consequently, the aim of this study is to investigate the dose–response relationship of long-lasting static stretching on MSt. A total of 70 active participants (f = 30, m = 39; age: 27.4 ± 4.4 years; height: 175.8 ± 2.1 cm; and weight: 79.5 ± 5.9 kg) were divided into three groups: IG1 and IG2 both performed unilateral stretching continuously for one (IG1) or two hours (IG2), respectively, per day for six weeks, while the CG served as the non-intervened control. MSt was determined in the plantar flexors in the intervened as well as in the non-intervened control leg to investigate the contralateral force transfer. Two-way ANOVA showed significant interaction effects for MSt in the intervened leg (ƞ^2^ = 0.325, *p* < 0.001) and in the contralateral control leg (ƞ^2^ = 0.123, *p* = 0.009), dependent upon stretching time. From this, it can be hypothesized that stretching duration had an influence on MSt increases, but both durations were sufficient to induce significant enhancements in MSt. Thus, possible applications in rehabilitation can be assumed, e.g., if no strength training can be performed, atrophy could instead be reduced by performing long-lasting static stretch training.

## 1. Introduction

Increasing strength capacity is of high importance to the prevention [1] and rehabilitation of orthopedic indications [2,3]. Mechanical tension is an important stimulus to achieving an increase in maximal strength (MSt), which is commonly induced by strength training [4,5,6]. While low intensities seem to be sufficient to induce hypertrophy [7,8]**,** a load intensity of 60–80% of the one-repetition maximum (1RM) is generally recommended as an appropriate method to achieve both MSt and muscle hypertrophy [4,9]. From this, it can be hypothesized that high intensities seem to be more beneficial to achieve improvements in MSt [8,10]. Results regarding animal research from between 1970–1995 show that long-lasting static stretching can induce sufficient mechanical stress, leading to muscle hypertrophy and increases in MSt [11,12,13,14], too. Assuming a transferability to humans, static stretching could be used in the rehabilitation of injury-related immobilization of lower limb muscles, as this usually results in significant atrophy and strength loss [15,16]. To date, there are some studies that have investigated the effects of short duration stretch training for several weeks on the maximal strength of humans [17,18,19]. Many of these studies show a significant increase in MSt due to a long-term static stretching intervention [17,20,21]. Yahata et al. [22] found a significant increase in MSt of 6.4 ± 9.9% in the calf muscles within a five-week period by stretching twice a week with durations of 6 × 5 min using a stretching board. In addition, Kokkonen et al. [20], Nelson et al. [17] and Mizuno et al. [23] were able to achieve significant MSt increases of 15.3% to 32.4% due to stretch training for up to ten weeks, while other studies investigated the effects of a daily stretching routine on concentric peak torque and found significant increases of 9.3% [24] and 11% [16], respectively.

Cross-education effects are known from unilateral strength training [25,26,27]. Zhou [28] and Zhou et al. [29] suggested that adaptations in motor learning are a preferred explanation of cross-education effects by showing that the electrical stimulation on one leg results in strength increases in the contralateral leg. The authors hypothesized that the cross-training effects could be attributed to afferent modulations. Based on these suggestions, Nelson et al. [17] hypothesized that static stretching could also provide a sufficient stimulus to induce a contralateral force transfer due to its activation of afferents and the impact on MSt in the stretched limb. The authors confirmed their hypothesis by demonstrating an increase in maximal strength of up to 29% (d = 1.24) in the stretched leg, which was accompanied by a contralateral force transfer of 11% to the contralateral leg (d = 0.46) by inducing 4 × 30 s of stretching for three days per week for ten weeks. Furthermore, Caldwell et al. [30] showed that stretching the quadriceps twice daily for two weeks resulted in significant increases in MSt of 7.1% (d = 0.8) in the stretched leg and of 6.6% (d = 0.45) in the contralateral leg, while stretching once per day did not lead to a contralateral force transfer. Warneke et al. [31] also showed cross-educational effects in dynamic strength testing of 11.4% (*p* < 0.001) after stretching the plantar flexors for one hour, while no effects were obtained in isometric strength testing (1.4%, *p* = 0.46). Furthermore, jumping height was also increased in the stretched as well as in the contralateral leg with 13.7%, d = 1.28 and 13%, d = 1.01, respectively. This was confirmed by Panidi et al. [32] who showed improvements in jumping height of 27%, d = 0.78 and 17%, d = 0.46 in the stretched and unstretched control leg, respectively. However, Handel et al. [33] found no effects in the contralateral side from unilateral training.

Only one investigation has examined the long-term effects of long-lasting static stretching on MSt, showing significant improvements in MSt of about 16% in the plantar flexors by using a one-hour stretching protocol daily [31]. However, some investigations could not point out any significant changes in MCSA or maximal strength after several weeks of stretch training [18,22,34]. Inconsistencies in intensity and volume could explain the heterogeneity of the results in the listed investigations in regard to MSt gains following a stretching stimulus. Furthermore, Apostolopoulos et al. [35] pointed out the high relevance of high intensity in stretching interventions: at lower intensities, tension is compensated via elastic tissue instead of generating adequate muscle tension. Considering a dose–response relationship of stretching in animal experiments [36,37] and the results from Warneke et al. [31], it can be assumed that long-lasting stretching could lead to improved MSt capacity in humans, which can be seen as highly relevant in designing rehabilitation programs: “The therapeutic application of stretch should therefore be kept in mind when designing regimes for rehabilitation” [38]. Therefore, the aim of this longitudinal study was to investigate the effects of a stretching stimuli lasting one or two hours per day, respectively, on MSt in the plantar flexors with a bended knee joint, and to investigate whether a contralateral strength transfer can be induced via long-lasting stretching interventions.

## 2. Materials and Methods

To answer the research question, MSt in the plantar flexors was examined with a bended knee joint via unilateral isometric strength testing for both legs in pre- and post-test. Afterwards, a daily unilateral stretching intervention was performed for one or two hours using a stretching orthosis for six weeks.

### 2.1. Participants

The study procedure was approved by the ethics vote 2019-016 of the ethics committee of the Carl von Ossietzky University Oldenburg. Seventy active participants were recruited from sports study programs, sports clubs and gyms. They were divided into three groups (two experimental groups (IG1 and IG2), as well as one control group (CG). Characteristics of subjects are provided in Table 1. The differences in group size can be explained by the willingness of active participants to partake in the long-lasting intervention groups; especially in IG2 with a stretching duration of two hours per day. Participants were categorized as active if they performed two to three training sessions in a gym or team sport continuously for the past six months. They were instructed to continue with their previous training routine throughout the six-week intervention period; consequently, long-lasting stretching was performed in addition to existing routines. However, participants were not allowed to perform any separated calf muscle training within the six-week training intervention.

### 2.2. Testing Procedure

#### 2.2.1. Maximum Strength Measurement

**Figure 1:**
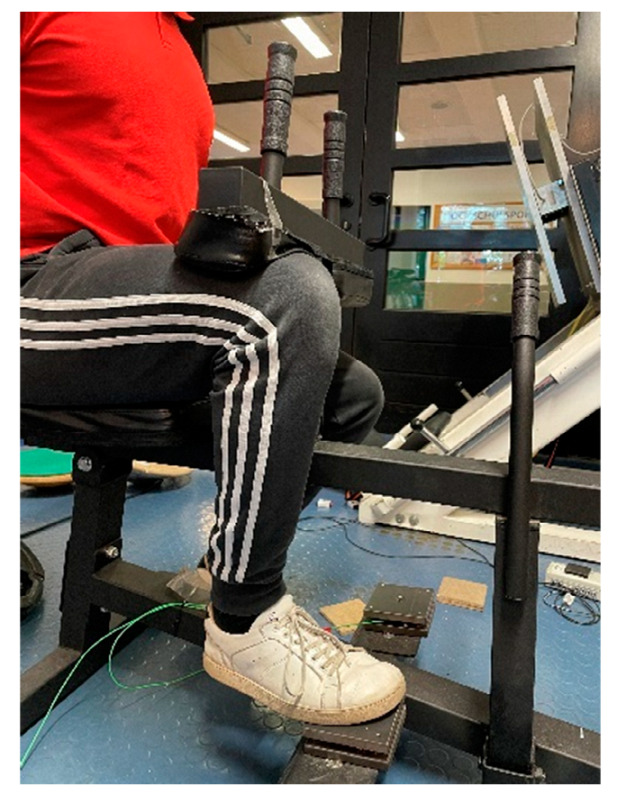
Calf-muscle-testing device (CMD) measuring the maximum isometric strength in pre- and post-test with force plates.

All participants performed a pre- and post-test. For all participants, the maximum isometric strength (MStiso) in the bended knee joint was recorded for both legs using unilateral testing (see Figure 1). For this purpose, the participant was instructed to perform a plantar flexion for three seconds with a maximum voluntary contraction against the pad of the measuring device in response to an acoustic signal. The seated calf raise machine was adjusted for each participant to achieve a 90° angle in the participant’s ankle and knee joints. Testing was performed until the force values stopped increasing with a minimum of five trials. The MStiso was determined in each case using a 10 × 10 cm force measurement platform in which force sensors Kistler Element 9251A with a resolution of 1.25 N, a pull-in frequency of 1000 Hertz and a measurement range of ±5000 N were installed. The vertical forces (Fz) were recorded. A Typ5009 Charge Amplifier and a 13-bit AD converter NI6009 were used. A calculation program (Carl von Ossietzky University Oldenburg) was used to illustrate the force–time curves of the vertical forces from the unfiltered raw data to provide an objective determination of MSt for further evaluation, and to rule out any artifacts that could affect the results. Reliability was determined with an ICC between the best and second-best value reached in each test, which were classified as high when the ICC = 0.994 and CV = 1.89%.

#### 2.2.2. Intervention

**Figure 2:**
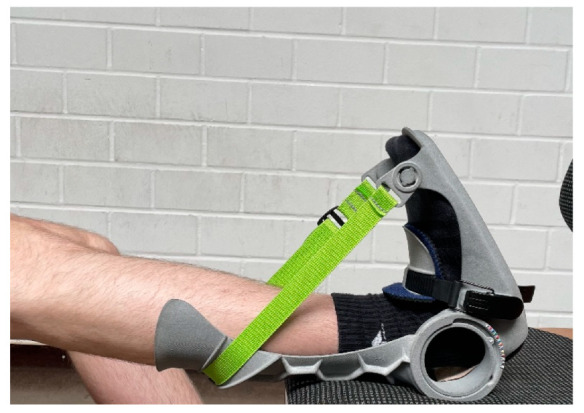
Orthosis used for calf-muscle stretching.

The intervention consisted of daily stretch training of the calf muscles, induced by wearing an orthosis developed for this purpose, which comprised an angle-measuring apparatus to quantify the stretching intensity (see Figure 2). Stretching was performed on only one leg to be able to investigate the cross-education effects. Thereby, the intervention was performed on the dominant leg. Group IG1 stretched for one hour per day and group IG2 stretched for two hours per day for seven days per week over a period of six weeks. Thus, a weekly stretching volume of seven hours in IG1 and fourteen hours in IG2 was performed. Daily stretch training was performed because similar animal experiments exhibited significant positive effects on MSt gains [11,12]. The orthosis had to be worn with the knee extended. Participants were instructed to sit with their back straight against a backrest while wearing the orthosis and to place their foot on an object that was the same height as the chair they were sitting on. In order to address the general problem of documenting the training intensity in stretching interventions, the angle of the ankle joint was set and controlled via the goniometer on the orthosis. The intensity was to be set by the participant so that the individual stretching pain corresponded to an eight on a scale of 1–10, where 1 represented no stretching pain and 10 was determined as the maximal tolerable stretching pain. The angle set for this purpose was read off by the participants and documented in the stretch diary.

### 2.3. Data Analysis

The analysis was carried out with SPSS 28. As previously performed by Caldwell et al. [30], a mixed-model ANOVA was performed for the collected parameters with variables tested for group, time and interaction group * time. Separate two-way ANOVAs with repeated measures were used for the three groups (IG2, IG1 and CG) to analyze the influence of static stretching on the intervened and the non-intervened leg. Another separate analysis was performed which included four groups, investigating the contralateral force transfer (intervened leg of IG1, non-intervened leg of IG1 and both legs of the CG; and intervened leg of IG2, non-intervened leg of IG2 and both legs of the CG). The Scheffé test was used as a post-hoc test. Effect sizes are presented as Eta squared (ƞ^2^) and categorized as: small effect ƞ^2^ < 0.06, medium effect ƞ^2^ = 0.06–0.14, large effect ƞ^2^ > 0.14 [39]. Furthermore, effect sizes for increases in MSt were calculated and provided [39]. Power analysis was performed by using post-hoc power analysis via G-Power.

## 3. Results

The normal distribution of data was ensured by using the Shapiro–Wilk test. All participants who participated in the pre-tests completed the study. No problems were reported on the intervention and the use of the orthosis. The prescribed wearing durations were fulfilled by all participants. Levene’s test for variance homogeneity yielded *p* > 0.05.

### 3.1. Overall Statistics

Table 2 provides the mean (M) and standard deviations (SD) for the pre- and post-test values of all included groups.

The results showed no significant group effect for the pre-test in the intervened leg with F_2.72_ = 0.96, *p* = 0.39 with ƞ^2^ = 0.026, as well as in the non-stretched leg with F_2.72_ = 0.72, *p* = 0.49, ƞ^2^ = 0.02. In the intervened groups, a significant post-test effect was observed with F_2.72_ = 8.08, *p* < 0.001 and ƞ^2^ = 0.18, while there was no significant group effect for post-test values in the contralateral leg F_2.72_ = 2.54, *p* = 0.086, ƞ^2^ = 0.07.

In the overall statistics, the two-way ANOVA revealed high effects for the time effect (F_1.69_ = 48.48; ƞ^2^ = 0.2.75), as well as for the interaction effect group * time (F_2.68_ = 10.06; ƞ^2^ = 0.28) with *p* < 0.001. The mean value in IG1 increased by 14.2% (*p* < 0.001, d = 0.51) from pre-test to post-test, and by 22.3% (*p* < 0.001, d = 0.91) in IG2; the CG did not change significantly, changing by 1.9% (*p* = 0.45). The mean value in IG1 increased by 5.5% (*p* = 0.024, d = 0.18) from pre-test to post-test, and by 10.9% (*p* = 0.011, d = 0.36) in IG2; the control group did not change significantly, changing by 1.1% (*p* = 0.45).

The group differences determined by the Scheffé test showed significant differences between IG1il and the CG (*p* = 0.003–0.004), as well as between IG2il and the CG (*p* < 0.001). No significant differences could be determined between IG1il and IG1cl (*p* = 0.392), or between IG2il and IG2cl (*p* = 0.41). Furthermore, the Scheffé test showed no significant differences for IG1cl and the control group (*p* = 0.56–0.60), or between IG2cl and the control group (*p* = 0.14–0.16).

For a more precise analysis that considers the differences between separate legs, further analysis was performed for the intervened legs of IG1 and IG2 compared to CGl, and the non-intervened leg compared to CGr.

### 3.2. Analysis of Maximum Strength Tests of the Intervened Leg

The two-way ANOVA revealed high effects for the time effect (F_1.69_ = 54.245; ƞ^2^ = 0.430, d = 1.74), as well as for the interaction effect group * time (F_2.68_ = 18.494; ƞ^2^ = 0.325, d = 1.39) with *p* < 0.001. The mean value in IG1 increased by 14.2% (*p* < 0.001, d = 0.51) from pre-test to post-test, and by 22.3% (*p* < 0.001, d = 0.91) in IG2; the CG did not change significantly, changing by 1.9% (*p* = 0.45). The group differences determined by the Scheffé test exhibited significant differences between the mean of IG1 and the CG (*p* < 0.001), and IG2 and the CG (*p* < 0.001). No significant differences were found between IG1il and IG2il (*p* = 0.23).

### 3.3. Analysis of Maximum Strength Tests of the Non-Intervened Leg

Descriptive data of MStiso testing of the intervened legs of IG1il and IG2cl, as well as the left legs of the control group are provided in Table 2.

The two-way ANOVA revealed medium effects for the time effect (F_1.69_ = 10.761; *p* = 0.002; ƞ^2^ = 0.130, d = 0.77) and the interaction effect group * time (F_2.68_ = 5.063;*p* = 0.009; ƞ^2^ = 0.123, d = 0.749). The mean value in IG1cl increased by 5.5% (*p* = 0.024, d = 0.18) from pre-test to post-test, and by 10.9% (*p* = 0.011, d = 0.36) in IG2; the control group did not change significantly, changing by 1.1% (*p* = 0.45). The Scheffé test showed a significant difference in mean differences, only for IGcl2 vs. the CG (*p* = 0.014).

**Figure 3:**
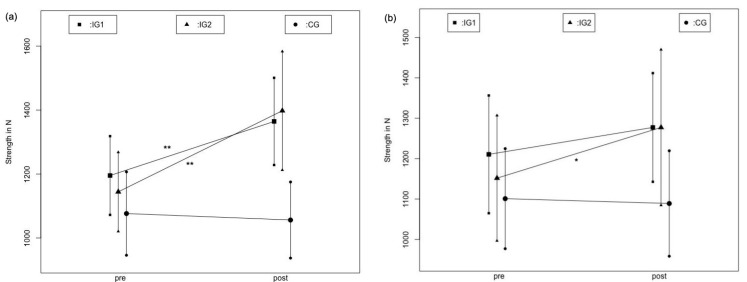
Comparison of maximum strength in pre- to post-test between IG1il, IG2il and CG (**a**) as well as between IG1cl, IG2cl and CGr (**b**). ** = *p* < 0.001, * = *p* < 0.05 for difference to control group.

Figure 3 shows the mean value curve of the maximum strength values in intervention groups, as well as in the control group in pre- and post-testing for the stretched leg. Values in IG1 and IG2 represent the stretched leg. Values in the CG represent the left leg of the control group.

### 3.4. Analysis of the Stretched Leg versus the Non-Stretched Leg within One Group to Examine the Contralateral Force Transfer

For IG1, the two-way ANOVA revealed a significant moderate time effect with F_1.116_ = 17.78, *p* < 0.001, ƞ^2^ = 0.13 and a significant high interaction effect group * time with F_3.116_ = 12.84, *p* < 0.001, ƞ^2^ = 0.25. The Scheffé test showed significant differences between IG1il and both legs of the control group (*p* < 0.001), but no significant difference between IG1il and IG1cl (*p* = 0.062). No significant differences between IG1cl and both legs of the CG were determined (*p* = 0.96–0.156). For IG2, the two-way ANOVA revealed a significant moderate time effect with F_1.98_ = 28.95, *p* < 0.001, ƞ^2^ = 0.23 and a significant high interaction effect group * time with F_3.78_ = 15.48, *p* < 0.001, ƞ^2^ = 0.32. The Scheffé test showed significant differences between IG2il and both legs of the control group (*p* < 0.001), but no significant difference between IG2il and IG2cl (*p* = 0.14). A significant difference between IG2cl and both legs of the CG were also determined (*p* = 0.02–0.033).

**Figure 4:**
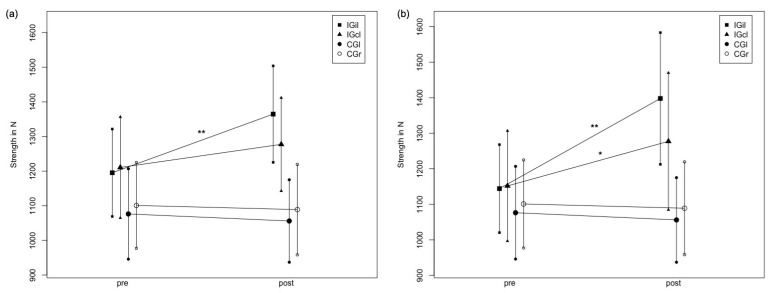
Illustrating the mean value curve of the maximum strength values in IG1il, IG1cl and both groups of CG (CGl and CGr) (**a**) as well as the mean value curve of the maximum strength values in IG2il, IG2cl and both groups of CG (**b**). ** = *p* < 0.001, * = *p* < 0.05 for difference to control group.

The results are graphically illustrated in Figure 4, presenting changes in MStiso from the pre- to post-test for IG1il and cl, and both test groups for the CG and for IG2il and cl, and both test groups for the CG.

Post-hoc analysis for F-tests of G-Power were calculated as 1 − β = 42.00% for the lowest effect size with ƞ^2^ = 0.123, and 1 − β = 99.99% for the highest effect size with ƞ^2^ = 0.430, and with α = 0.05 for the three groups and two time points for the interaction.

## 4. Discussion

Stretch training of one and two hours, respectively, resulted in significant increases in MStiso (*p* < 0.001 in both groups), while no significant increases in MStiso were measured in the CG (*p* = 0.45). Two hours of daily stretch training resulted in an average MStiso increase of 22.3% in the intervened leg, and a contralateral force transfer to the non-intervened leg of 10.9%, *p* = 0.011 in the control leg. Stretching the calf muscle for one hour daily resulted in a 14.2% (*p* < 0.001) increase in average MSt in the calf muscles of the intervened leg and a 5.5% (*p* = 0.024) increase in MSt in the control leg. However, the statistical analysis revealed no significant difference between IG2il and IG1il (*p* = 0.23). Furthermore, no statistically significant difference was determined between IG1cl and IG2cl (*p* = 0.489), but it was determined between IG2cl and the CG. The results were confirmed by investigating the contralateral force transfer within the intervention groups, showing no significant difference between IG1il and IG1cl (*p* = 0.062) and between IG1cl and CGs (*p* = 0.156–0.96). However, IG2 showed significant differences between IG2il and CGs (*p* < 0.001) and between IG2cl and CGs (*p* = 0.02–0.033). The results indicate that stretching one leg for two hours per day resulted in significant strength increases in the stretched as well in the non-stretched leg (i.e., contralateral force transfer), while one hour of daily stretching showed significant improvements in the stretched leg without a statistically significant force transfer.

The aim of this study was to investigate if doubling the stretching time would also lead to significantly higher increases in MSt capacity; however, although there were increases in MSt of 22.3% due to two-hour daily stretching compared to 14.2% due to one-hour daily stretching for six weeks, the difference failed to be statistically significant. Thus, this hypothesis must be declined. It is known from animal studies that longer stretching times per training session led to higher increases in muscle mass with a dose–response relationship [37]. Previous research on daily long-lasting static stretching showed significant increases in MSt measured in the extended knee joint, comparable to those in the present study. Since Warneke et al. [31] showed significant hypertrophy in the plantar flexors due to long-lasting stretching intervention, which could possibly be attributed to stretch-induced mechanical tension, leading to hypertrophy and MSt increases, a general transferability of the effects observed in animal studies to humans could be hypothesized. The hypothesis of mechanical-induced structural changes are confirmed by the results of a variety of animal studies [40], showing significant increases in muscle mass, the muscle cross-sectional area and the fiber cross-sectional area due to chronic stretching interventions: “It is Stretch that causes the Hypertrophy of Muscle” ([41] p. 93). However, increased MSt in the first weeks of training are commonly related to neuronal aspects [42,43], while morphological changes might be of minor relevance. The measured contralateral force transfer can also be seen as confirmation of the inclusion of neuronal aspects in the stretching-induced MSt increases. Zhou et al. [29] referred to different methods of peripheral stimulation, e.g., electrical stimulation or vibration training to induce cross-educational effects; however, the underlying mechanisms remain unclear. Based on the current literature, the authors hypothesize that peripheral sensory inputs might play an important role in inducing contralateral force transfer effects. Consequently, it is possible that long-lasting stretching provides sufficient peripheral stimulation in the intervened leg (e.g., via nociception) that leads to cross-education effects. Caldwell et al. [30] also referred to tension as the mechanism behind MVC improvements following stretching interventions. However, the contralateral force transfer may be attributed to changes in neuronal activity, which could possibly be attributed to afferences [28] due to the involvement of muscle spindles [33], while Caldwell et al. [30] referred to the possibility of active contraction against the stretching device. A high involvement of neuronal activity due to stretching can be assumed as many non-local effects can be observed via stretching interventions [44,45]. The results show that there was a statistically significant contralateral strength transfer exclusively in the two-hour stretching group, while one hour of static stretching did not reach statistical significance when compared to the control group.

Nelson et al. [17] found a contralateral force transfer from stretching intervention over a ten-week training period in humans. They were able to determine an 11% increase in the control leg when stretching three times a week. While Nelson and colleagues [17] assumed the cause of the MSt increase in the contralateral leg to be the stabilization of the body during the stretch training, such an effect can be excluded in the present work because the stretching intervention was performed in a seated position. The comparatively high MSt increases of 29% in the intervention leg as well as 11% in the control leg reported by Nelson et al. [17]**,** as well as in Kokkonen et al. [20]**,** can possibly be attributed to the conditional training status of the participants, if the authors attribute the MSt increases in the non-intervened leg to the stabilization of the body during the stretch training. In trained participants, no MSt increase would be expected due to the stabilizing activity during stretch training. Additionally, there are investigations showing that training with low load intensities, but with the addition of blood-flow restriction, led to muscular hypertrophy as well as increases in MSt [7,46]. Because participants in our investigation reported initial numbness after approximately 10–15 min, blood hypoxia combined with a mechanical stretch stimulus could be hypothesized to be the underlying mechanism for adaptations in MSt, similar to blood flow restriction training. In animal studies, Hotta et al. [47] demonstrated that 30 min of stretching per day resulted in a significant enhancement in blood supply to the stretched musculature as during the stretch, the blood inflow to the muscle was highly inhibited. In further studies, the influence of blood flow and VO_2_ to the working muscle should be investigated regarding the improvement in strength capacity.

In general, the results were limited because gender was not balanced in each group. Therefore, gender specific differences could not be examined in the context of this study, especially because there were just three females in IG2. However, the aim of this study was not to investigate gender-related differences regarding stretching-induced improvements in MSt. Since it is well accepted that stretch training could lead to increases in flexibility in general [48,49], and Warneke et al. [31] showed significant improvements in flexibility due to a similar stretching intervention, we did not examine the influence of stretching on flexibility in this study. It was not possible to include equal sample sizes in the study as not all participants were willing to join the two-hour stretching group. It could be hypothesized that differences between IG1 and IG2 did not reach the level of significance because of unequally distributed groups and a comparably low sample size in IG2, including high SDs. Further research should include higher sample sizes. Furthermore, to investigate the dose–response relationship, which is known from animal studies, the investigation of different stretching durations is requested. Since neither imaging techniques (such as sonography or magnetic resonance imaging) were used to assess muscle hypertrophy, nor were EMG measurements conducted to quantify changes in neuronal innervation, the physiological factors of the increased MSt, as well as the cross-education effects, remain unclear. As Manca et al. [50] highlighted a variety of possible explanations and Zhou et al. [29] referred to the need for clarification regarding the underlying mechanisms of contralateral effects due to unilateral peripheral stimulation, further studies should include additional measuring procedures to gain more insights.

## 5. Conclusions

In general, increases in MSt due to long-lasting static stretching of one or two hours in both the stretched and the non-stretched contralateral muscle can possibly be attributed to neuronal adaptations via changes in innervation of the central nervous system, or morphological adaptations due to changes in muscle architecture and muscle mass [31,51]. Since the intervention period lasted six weeks, and observed increases in MSt can be majorly attributed to changes in neuronal adaptations in the first few weeks of a strength training [42] and a contralateral strength transfer by using two hours of static stretching, improvements in MSt are suggested to be primarily attributed to neuronal adaptations. Further research is needed to obtain deeper insights (e.g., EMG measurements and magnetic resonance imaging) on the increases in MSt due to long-lasting static stretching interventions.

## 6. Practical Application

The results show that stretching can lead to an improvement in MSt when performed for a sufficient duration and under a sufficient intensity, leading to adequate muscle tension. If the aim is to induce increases in MSt in the contralateral leg, a long stretching duration (>1 h) seems to be required. Especially in the case of lower extremity injuries that lead to immobilization, and thus to a loss of MSt, mobility and the muscle cross-sectional area, this training method and implementation via a calf-stretching orthosis appears to be useful. The early use of this training method could counteract immobilization-related loss of strength and mobility. Therefore, the use of long-lasting stretching interventions in the early phase of immobilization should be tested. In addition to applications in rehabilitation, there is a potential use for astronauts, since time in a weightless environment results in atrophy effects and conventional strength training with external weights seems unfeasible in implementation [52,53].

## Figures and Tables

**Table 1:** Characteristics of subjects.

Group	N	Age (in Years)
Total	70	24.1 ± 3.5
IG1	25 (f = 7; m = 18)	23.4 ± 4.7
IG2	15 (f = 3; m = 12)	27.2 ± 5.3
CG	30 (f = 14; m = 16)	24.6 ± 3.8

**Table 2:** Descriptive statistics of the maximum strength values in intervention groups as well as in control group in pre- and post-testing for the stretched leg.

Group	Pre-Test (M ± SD) in N	Post-Test (M ± SD) in N
IG1il	1195.3 ± 321.09	1364.54 ± 355.43
IG1cl	1210.6 ± 371.8	1277.2 ± 343.2
IG2il	1144.2 ± 244.7	1397.9 ± 366.5
IG2cl	1151.7 ± 306.5	1277.2 ± 380.8
CGl	1076.3 ± 364.5	1056.0 ± 332.7
CGr	1100.9 ± 346.1	1088.9 ± 364.8

## Data Availability

Original data can be provided from corresponding author due to reasonable request.
